# Checklist of the continental fishes of the state of Chiapas, Mexico, and their distribution

**DOI:** 10.3897/zookeys.632.9747

**Published:** 2016-11-16

**Authors:** Ernesto Velázquez-Veláquez, Jesús Manuel López-Vila, Adán Enrique Gómez-González, Emilio Ismael Romero-Berny, Jorge Luis Lievano-Trujillo, Wilfredo A. Matamoros

**Affiliations:** 1 Museo de Zoología, Instituto de Ciencias Biológicas, Universidad de Ciencias y Artes de Chiapas. Libramiento Norte Poniente No. 1150, Colonia Lajas Maciel, C.P. 29039. Tuxtla Gutiérrez, Chiapas, México; 2 Posgrado en Ciencias Biológicas, Instituto de Biología, Universidad Nacional Autónoma de México; Tercer circuito s/n, Ciudad Universitaria, Copilco, Coyoacán. A.P. 70-153, C.P. 04510, Ciudad de México, México; 3 Centro de Investigaciones Costeras, Instituto de Ciencias Biológicas, Universidad de Ciencias y Artes de Chiapas. Calle Juan José Calzada s/n, Colonia Evolución, C.P. 30500. Tonalá, Chiapas, México

**Keywords:** Distribution, endemism, fish diversity, ichthyology, southern Mexico

## Abstract

An updated checklist of the distribution of fishes that inhabit the continental waters of the Mexican state of Chiapas is presented. The state was compartmentalized into 12 hydrological regions for the purpose of understanding the distribution of fish fauna across a state with large physiographic variance. The ichthyofauna of Chiapas is represented by 311 species distributed in two classes, 26 orders, 73 families, and 182 genera, including 12 exotic species. The families with the highest number of species were Cichlidae, Poeciliidae, Sciaenidae, Carangidae, Ariidae, Gobiidae, and Haemulidae. This study attempts to close gaps in knowledge of the distribution of ichthyofauna in the diverse hydrological regions of Chiapas, Mexico.

## Introduction

The hydrological wealth of Chiapas is manifested through its 72 perennial rivers and abundant streams, lakes, and ponds. The presence of large hydroelectric dams has significantly increased the surface area of the state’s bodies of water (Velasco-Colín 1976). Chiapas has a coastline of 270 km and more than 70,000 hectares of estuaries and coastal lagoons (Contreras-Espinosa 2010), which favors the presence of rich fish diversity (Velasco-Colín 1976, Lozano-Vilano and Contreras-Balderas 1987, Rodiles-Hernández et al. 2005). Much of the state is located in the Usumacinta ichthyographic province/area of endemism (Miller et al. 2005, Matamoros et al. 2015), which means that its continental waters host a high number of endemic species, making Chiapas a freshwater biodiversity hotspot (Hudson et al. 2005, Matamoros et al. 2015).

Several attempts have been made to record continental water fish diversity in Chiapas through numerous works such as checklists, annotated checklists, books and scattered records in the literature (e.g. Velasco-Colín 1976, Lozano-Vilano and Contreras-Balderas 1987, Lazcano-Barrero and Vogt 1992, Tapia-García et al. 1998, Rodiles-Hernández et al. 1999, Rodiles-Hernández 2005, Rodiles-Hernández et al. 2005, 2013, Lozano-Vilano et al. 2007, González-Díaz et al. 2008, Espinosa-Pérez et al. 2011, Velázquez-Velázquez et al. 2013, Gómez-González et al. 2012, 2015). The first comprehensive publication on continental fishes of Chiapas was made by Velasco-Colín (1976), who reported 74 species distributed across 28 families. He also included brief information about the ecology, biology and distribution of several species and, in some cases, added relevant fishing information.

Subsequently Lozano-Vilano and Contreras-Balderas (1987) published an annotated checklist in which they registered 135 species belonging to 38 families in the state’s continental waters. In addition to an increase in the number of data records, for the first time the distribution of fishes was associated with seven of the state’s physiographic regions. Eighteen years later Rodiles-Hernández (2005) and Rodiles-Hernández et al. (2005) recorded 205 species in 44 families and 207 species in 45 families respectively. In the first study, distributions were reported at the level of the two main Chiapas river basins, the Grijalva-Usumacinta and the Coast of Chiapas, whereas, in the second study, the distributional geographic units were the Atlantic and the Pacific slope. Velázquez-Velázquez et al. (2013) was the last published attempt to summarize continental fishes of Chiapas. They reported 262 species across 57 families, and once again the geographic distribution units were the Grijalva and the Usumacinta River basins and the coast of Chiapas.

Two interesting trends emerge about the continental fishes of Chiapas. First, the number of recorded species has continued to increase over time likely due to an increase in sampling localities, implementation of new sampling techniques, new records and species descriptions. The second trend is related to the geographic units in which the state has been divided. For instance, Lozano-Vilano and Contreras-Balderas (1987) divided the state into seven physiographic regions, based on terrestrial relief. Most studies used broad delineations limited to the three major hydrologic regions (coast of Chiapas and the Grijalva and Usumacinta River basins) masking detailed information on finer distributional patterns like localized endemism and drainage interconnections.

Therefore, the aim of this study is to provide an updated checklist of the continental fishes of Chiapas, including distribution data, based on extensive literature research and complemented with material deposited in the ichthyological collection of the Museum of Zoology at the University of Arts and Sciences of Chiapas (MZ-P-UNICACH). For the first time, we use finer scale geographic divisions for the state, implemented at the sub-basin level, following the National Institute of Statistics and Geography (INEGI 2010).

## Materials and methods

The bulk of records came from the material of 204 species deposited in the ichthyological collection of the MZ-P-UNICACH Museum of Zoology (MZ-P-UNICACH, SEMARNAT: CHIS-PEC-210-03-09). In addition, we performed an extensive literature review for records of continental fishes of Chiapas. The checklists previously published by Lozano-Vilano and Contreras-Balderas (1987), Rodiles-Hernández (2005), Rodiles-Hernández et al. (2005), Espinosa-Pérez et al. (2011), and Velázquez-Velázquez et al. (2013) were taken as the basis for this work and were supplemented with publications by Lazcano-Barrero and Vogt (1992), Tapia-García et al. (1998), Rodiles-Hernández et al. (1999), Lozano-Vilano et al. (2007) and Gómez-González et al. (2012, 2015) who developed lists for particular regions of the state. We also included Castro-Aguirre et al. (1999) and Miller et al. (2005).

Species were systematically arranged by order and family following Nelson (2006). Genera and species were arranged alphabetically; scientific names and authorities were corroborated following Eschmeyer et al. (2016). Tolerance to salinity was based on Myers (1938).

**Figure 1.**
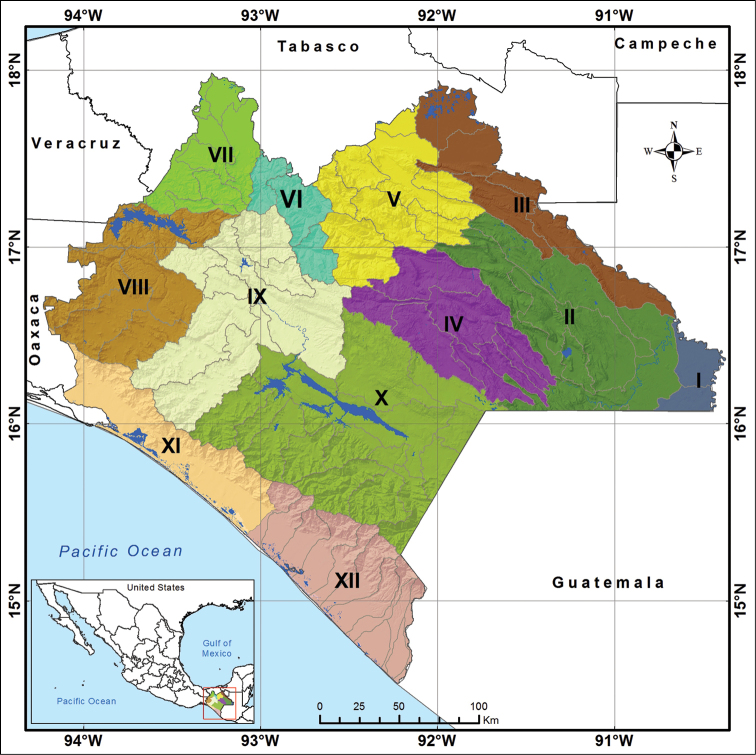
Geographical units for the study of the distribution of the fish fauna of the state of Chiapas: **I** (Usumacinta-Chixoy) **II** (Usumacinta-Lacantún) **III** (Usumacinta-Catazajá) **IV** (Usumacinta-Jataté) **V** (Grijalva-Tulijá) **VI** (Grijalva-Teapa) **VII** (Grijalva-Peñitas) **VIII** (Grijalva-Malpaso), IX (Grijalva-Chicoasén) **X** (Grijalva-La Angostura) **XI** (Costa-Itsmo) **XII** (Costa-Soconusco).

The 12 geographical units for Chiapas (Figure 1) were utilized to determine the distribution of each species across the state. These 12 units were based on existing hydrological sub-basins of the state (INEGI 2010). The main rivers, ponds, lakes and coastal lagoons of each sub-basin are listed in Table 1.

**Table 1.** Geographic units utilized to study the distribution of the fish fauna of Chiapas and sub-basins that form them.

Hidrological region	Basin	Sub-basin	Geographic unit
COSTA DE CHIAPAS	R. SUCHIATE AND OTHERS	R. Suchiate	**Costa-Soconusco**
		R. Cozoloapan	
		R. Cahuacán	
		Puerto Madero	
		R. Coatán	
		R. Huehuetán	
	R. HUIXTLA AND OTHERS	R. Huixtla	
		R. Despoblado	
		L. del Viejo y Tembladeras	
		R. Cacaluta	
		R. Sesecapa	
		R. Novillero	
	R. PIJIJIAPAN AND OTHERS	R. Margaritas y Coapa	**Costa-Istmo**
		R. Pijijiapan	
		R. San Diego	
		El Porvenir	
		R. Jesús	
		L. de la Joya	
	MAR MUERTO	R. Zanatenco	
		Mar Muerto	
		R. La Punta	
		R. Las Arenas	
		R. Tapanatepec	
GRIJALVA - USUMACINTA	R. USUMACINTA	R. Usumacinta	**Usumacinta-Catazajá**
		R. Chacamax	
		R. Chacaljáh	
	R. CHIXOY	R. Chixoy	**Usumacinta-Chixoy**
		R. Negro	
	R. GRIJALVA - VILLAHERMOSA	R. Viejo Mezcalapa	**Grijalva-Peñitas**
		R. Mezcalapa	
		R. Tzimbac	
		R. Zayula	
		R. Platanar	
		R. Paredón	
		R. Pichucalco	
		R. Tacotalpa	
		R. Samaria	
		R. de la Sierra	**Grijalva-Teapa**
		R. Almendro	
		R. Plátanos	
		R. Chacté	**Grijalva.Tulijá**
		R. Puxcatán	
		R. Macuspana	
GRIJALVA - USUMACINTA		R. Shumulá	
		R. Yashijá	
		R. Tulijá	
		R. Bascá	
		R. Chilapa	
	R. GRIJALVA - TUXTLA GUTIÉRREZ	P. Nezahualcóyotl	**Grijalva-Malpaso**
		R. La Venta	
		R. Encajonado	
		R. Cintalapa	
		R. de Zoyatenco	
		R. Alto Grijalva	**Grijalva-Chicoasén**
		R. Hondo	
		R. Chicoasén	
		R. Suchiapa	
		Tuxtla Gutiérrez	
		El Chapopote	
		R. Santo Domingo	
	R. GRIJALVA - LA CONCORDIA	P. La Angostura	**Grijalva-La Angostura**
		R. Selegua	
		R. Lagartero	
		R. Aguacatenco	
		R. San Pedro	
		R. La Concordia	
		R. Grande o Salinas	
		R. Aguazurco	
		R. San Miguel	
		R. Yahuayita	
		R. Zacualpa	
		R. Tapizaca	
		R. Comitan	
	R. LACANTÚN	R. Lacantún	**Usumacinta-Lacantún**
		R. Ixcán	
		R. Chajul	
		R. Lacanjá	
		R. San Pedro	
		L. Miramar	
		R. Perlas	
		R. Jataté	
		R. Azul	**Usumacinta-Jataté**
		R. Tzaconejá	
		R. Margaritas	
		R. Santo Domingo	
		R. Seco	
		R. Caliente	
		R. Euseba	

## Results

The continental fishes of the state of Chiapas are represented by two classes, 26 orders, 73 families, 182 genera and 311 species (Table 2), including 12 exotic species (*Ctenopharyngodon idella*, *Cyprinus carpio*, *Micropterus salmoides*, *Oncorhynchus mykiss*, *Oreochromis aureus*, *Oreochromis mossambicus*, *Oreochromis niloticus*, *Parachromis managuensis*, *Pterygoplichthys disjunctivus*, *Pterygoplichthys multiradiatus*, *Pterygoplichthys pardalis*, and *Tilapia zilli*). Only five species were endemic: the catfish *Lacantunia enigmatica*, the cichlids *Rocio ocotal* and *Thorichthys socolofi*, the killifish *Tlaloc hildebrandi* and the molly *Poecilia thermalis*. Based on species richness the most important families were: Cichlidae (35), Poeciliidae (29), Sciaenidae (18), Carangidae (17), Ariidae (16), Gobiidae (12), and Haemulidae (11). Almost all of these families, except the first two, contains peripheral species. These eight families represented 44.37% (138) of the state’s total species richness. Thirteen species are included in risk categories under Mexican law (NOM-059-SEMARNAT-2010; SEMARNAT 2010): *Poecilia sulphuraria* and *Tlaloc hildebrandi* are listed as endangered; *Priapella compressa*, *Thorichthys socolofi*, *Vieja hartwegi* and *Xiphophorus clemenciae* are listed as threatened; finally *Chiapaheros grammodes*, *Gambusia eurystoma*, *Hippocampus ingens*, *Potamarius nelsoni*, *Priapella intermedia*, *Rhamdia guatemalensis* and *Chuco intermedium* are listed as species under special protection. Based on general salinity tolerance, and excluding exotic species, 16 are primary freshwater fishes, 65 secondary freshwater fishes, and the rest of the species are peripheral (Table 2).

Of the 12 geographical units (Fig. 1), the region with the highest number of species was Costa-Itsmo with 174 species, followed by Costa-Soconusco with 153 species and the third was Usumacinta-Catazajá with 72 species. The region with the lowest recorded species was Usumacinta-Jataté with only 11 species. Numbers of species from other geographical units are presented in Table 2. Spatially, *Astyanax aeneus* and *Rhamdia guatemalensis* appeared in all regions within Chiapas. Other species with widespread distributions were *Poecilia sphenops* and the exotic cichlid *Oreochromis niloticus* (10 and 11 regions respectively). *Atherinella alvarezi*, *Brycon guatemalensis*, *Dorosoma anale*, *Dorosoma petenense*, and *Ictalurus meridionalis* were distributed in nine regions, while *Aplodinotus grunniens*, *Gambusia sexradiata*, *Ophisternon aenigmaticum*, *Parachromis managuensis*, *Poecilia mexicana*, *Pseudoxiphophorus bimaculatus*, and *Thorichtys helleri* were recorded in eight regions.

Eight marine species were newly recorded as species found in continental waters of Chiapas: *Acanthurus xanthopterus*, *Atherinella panamensis*, *Fistularia commersonii*, *Halichoeres dispilus*, *Nicholsina denticulata*, *Orthopristis chalceus*, *Stegastes flavilatus*, and *Sphyraena ensis*.

**Table 2.** Systematic list of the continental waters ichthyofauna of Chiapas. Ecological classification: PF (Primary Freshwater), SF (Secondary Freshwater), PF (Peripheral Vicarious), PC (Peripheral Catadromous), P (Peripheral), Ex (Exotic).

No	Taxon	Ecological classification	Grijalva- La Angostura	Grijalva-Chicoasén	Grijalva-Malpaso	Grijalva-Peñitas	Grijalva- Teapa	Grijalva-Tulijá	Usumacinta-Jataté	Usumacinta-Lacantún	Usumacinta- Chixoy	Usumacinta-Catazajá	Costa-Istmo	Costa-Soconusco
**Order Carcharhiniformes**														
**I Family Carcharhinidae**														
1	*Carcharhinus leucas* (Müller & Henle, 1839)	P											x	
2	*Carcharhinus limbatus* (Müller & Henle, 1839)	P											x	x
3	*Carcharhinus cerdale* Gilbert, 1898	P											x	
4	*Rhizoprionodon longurio* (Jordan & Gilbert, 1882)	P											x	
5	*Negaprion brevirostris* (Poey, 1868)	P											x	
**II Family Sphyrnidae**														
6	*Sphyrna tiburo* (Linnaeus, 1758)	P											x	
**Order Pristiformes**														
**III Family Pristidae**														
7	*Pristis pectinata* Latham, 1794	P											x	
8	*Pristis microdon* Latham, 1794	P											x	
**Order Rhinobatiformes**														
**IV Family Rhinobatidae**														
9	*Pseudobatos glaucostigma* (Jordan & Gilbert, 1883)	P											x	
**Order Myliobatiformes**														
**V Family Urotrygonidae**														
10	*Urotrygon aspidura* (Jordan & Gilbert, 1882)	P											x	
11	*Urotrygon chilensis* (Günther, 1872)	P											x	
12	*Urotrygon munda* Gill, 1863	P											x	
13	*Urotrygon nana* Miyake & McEachran, 1998	P											x	
14	*Urotrygon rogersi* (Jordan & Starks, 1895)	P											x	
**VI Family Dasyatidae**														
15	*Hypanus longus* (Garman, 1880)	P											x	x
16	*Himantura pacifica* (Beebe & Tee-Van, 1941)	P											x	x
**VII Family Myliobatidae**														
17	*Aetobatus laticeps* Gill, 1865	P											x	x
**VIII Family Rhinopteridae**														
18	*Rhinoptera steindachneri* Evermann & Jenkins, 1891	P											x	x
**Order Lepisosteiformes**														
**IX Family Lepisosteidae**														
19	*Atractosteus tropicus* Gill, 1863	PF				x				x	x	x	x	x
**Order Elopiformes**														
**X Family Elopidae**														
20	*Elops affinis* Regan, 1909	P											x	x
**XI Family Megalopidae**														
21	*Megalops atlanticus* Valenciennes, 1847	P				x						x		
**Order Albuliformes**														
**XII Family Albulidae**														
22	*Albula esuncula* (Garman, 1899)	P											x	
**Order Anguilliformes**														
**XIII Family Ophichthidae**														
23	*Myrichthys xysturus* (Jordan & Gilbert, 1882)	P											x	
24	*Ophichthus zophochir* Jordan & Gilbert, 1882	P											x	x
**Order Clupeiformes**														
**XIV Family Pristigasteridae**														
25	*Pliosteostoma lutipinnis* (Jordan & Gilbert, 1882)	P											x	
26	*Odontognathus panamensis* (Steindachner, 1876)	P											x	
27	*Opisthopterus dovii* (Günther, 1868)	P												x
**XV Family Engraulidae**														
28	*Anchoa argentivittata* (Regan, 1904)	P											x	
29	*Anchoa curta* (Jordan & Gilbert, 1882)	P											x	x
30	*Anchoa ischana* (Jordan & Gilbert, 1882)	P											x	x
31	*Anchoa lucida* (Jordan & Gilbert, 1882)	P											x	x
32	*Anchoa mitchilli* (Valenciennes, 1848)	P										x		
33	*Anchoa mundeola* (Gilbert & Pierson, 1898)	P											x	x
34	*Anchoa walkeri* Baldwin & Chang, 1970	P											x	
35	*Anchoa starksi* (Gilbert & Pierson, 1898)	P											x	x
36	*Anchovia macrolepidota* (Kner, 1863)	P											x	x
**XVI Family Clupeidae**														
37	*Dorosoma anale* Meek, 1904	P (V)	x	x	x	x	x	x		x	x	x		
38	*Dorosoma petenense* (Günther, 1867)	P (V)	x	x	x	x	x	x		x	x	x		
39	*Harengula thrissina* (Jordan & Gilbert, 1882)	P											x	
40	*Lile gracilis* Castro-Aguirre & Vivero, 1990	P											x	x
41	*Lile nigrofasciata* Castro-Aguirre, Ruiz-Campos & Balart, 2005	P											x	x
42	*Opisthonema libertate* (Günther, 1867)	P											x	x
43	*Opisthonema medirastre* Berry & Barret, 1964	P											x	
**Order Gonorynchiformes**														
**XVII Family Chanidae**														
44	*Chanos chanos* (Forsskål, 1775)	P											x	x
**Order Cypriniformes**														
**XVIII Family Cyprinidae**														
45	*Ctenopharyngodon idella* (Valenciennes, 1844)^Ex^	Ex				x	x	x		x	x	x		
46	*Cyprinus carpio* (Linnaeus, 1758)^Ex^	Ex	x	x				x				x		
**XIX Family Catostomidae**														
47	*Ictiobus meridionalis* (Günther, 1868)	PF			x	x	x	x		x	x	x		
**Order Characiformes**														
**XX Family Characidae**														
48	*Astyanax aeneus* (Günther, 1860)	PF	x	x	x	x	x	x	x	x	x	x	x	x
49	*Bramocharax* sp.	PF						x		x	x	x		
50	*Brycon guatemalensis* Regan, 1908	PF	x	x	x	x	x	x		x	x	x		
51	*Hyphessobrycon compressus* (Meek, 1904)	PF				x				x	x	x		
52	*Roeboides bouchellei* Fowler, 1923	PF											x	x
**Order Siluriformes**														
**XXI Family Lacantuniidae**														
53	*Lacantunia enigmatica* Rodiles-Hernández, Hendrickson & Lundberg, 2005	PF								x				
**XXII Family Loricariidae**														
54	*Pterygoplichthys disjunctivus* (Weber, 1991) ^Ex^	Ex										x		
55	*Pterygoplichthys multiradiatus* (Hancock, 1828) ^Ex^	Ex										x		
56	*Pterygoplichthys pardalis* (Castelnau, 1855) ^Ex^	Ex				x	x	x		x		x		
**XXIII Family Heptapteridae**														
57	*Rhamdia guatemalensis* (Günther, 1864)	PF	x	x	x	x	x	x	x	x	x	x	x	x
58	*Rhamdia laluchensis* Weber, Allegrucci & Sbordoni, 2003	PF			x									
59	*Rhamdia laticauda* (Kner, 1858)	PF			x	x	x	x		x	x	x		
60	*Rhamdia parryi* Eigenmann & Eigenmann, 1888	PF											x	
**XXIV Family Ictaluridae**														
61	*Ictalurus meridionalis* (Günther, 1864)	PF	x	x	x	x	x	x		x	x	x		
**XV Family Ariidae**														
62	*Bagre panamensis* (Gill, 1863)	P												x
63	*Bagre pinnimaculatus* (Steindachner, 1876)	P												x
64	*Cathorops dasycephalus* (Günther, 1864)	P											x	
65	Cathorops cf. fuerthii	P												x
66	*Cathorops kailolae* Marceniuk & Betancur-R., 2008	P (V)		x	x	x				x	x	x		
67	*Cathorops liropus* (Bristol, 1897)	P											x	x
68	*Cathorops steindachneri* (Gilbert & Starks, 1904)	P											x	x
69	*Notarius kessleri* (Steindachner, 1876)	P												x
70	*Notarius planiceps* (Steindachner, 1876)	P											x	
71	*Notarius troschelii* (Gill, 1863)	P											x	
72	*Potamarius nelsoni* (Evermann & Goldsborough, 1902)	P (V)			x	x	x	x		x	x	x		
73	*Potamarius usumacintae* Betancourt-R. & Willink, 2007	P (V)								x	x	x		
74	*Sciades dowii* (Gill, 1863)	P												x
75	*Sciades felis* (Linnaeus, 1766)	P										x		
76	*Sciades guatemalensis* (Günther, 1864)	P											x	x
77	*Sciades seemanni* (Günther, 1864)	P											x	x
**Order Gymnotiformes**														
**XXVI Family Gymnotidae**														
78	*Gymnotus maculosus* Albert & Miller, 1995	PF												x
**Order Salmoniformes**														
**XXVII Family Salmonidae**														
79	*Oncorhynchus mykiss* (Walbaum, 1972)^Ex^	Ex		x										
**Order Aulopiformes**														
**XXVIII Family Synodontidae**														
80	*Synodus scituliceps* Jordan & Gilbert, 1881	P											x	x
**Order Batrachoidiformes**														
**XXIX Family Batrachoididae**														
81	*Batrachoides boulengeri* Gilbert & Starks, 1904	P											x	
82	*Batrachoides goldmani* Evermann & Goldsborough, 1902	P (V)			x	x	x	x		x	x	x		
83	*Batrachoides waltersi* Collette & Russo, 1981	P											x	x
84	*Porichthys greenei* Gilbert & Starks, 1904	P												x
**Order Mugiliformes**														
**XXX Family Mugilidae**														
85	*Agonostomus monticola* (Bancroft, 1834)	P (Ca)					x	x		x		x	x	x
86	*Joturus pichardi* Poey, 1860	P (Ca)								x		x		
87	*Mugil cephalus* Linnaeus, 1758	P											x	x
88	*Mugil curema* Valenciennes, 1836	P								x		x	x	x
89	*Mugil hospes* Jordan & Culver, 1895	P											x	x
**Order Atheriniformes**														
**XXXI Family Atherinopsidae**														
90	*Atherinella guatemalensis* (Günther, 1864)	P											x	x
91	*Atherinella alvarezi* (Díaz-Pardo, 1972)	P (V)	x	x	x	x	x	x		x	x	x		
92	*Atherinella panamensis* Steindachner, 1875	P											x	
93	*Atherinella schultzi* (Alvarez & Carranza, 1952)	P (V)				x				x	x	x		
94	*Membras gilberti* (Jordan & Bollman, 1889)	P											x	x
**Order Beloniformes**														
**XXXII Family Hemiramphidae**														
95	*Hyporhamphus mexicanus* Alvarez, 1959	P (V)			x	x	x	x		x	x	x		
96	*Hyporhamphus snyderi* Meek & Hildebrand, 1973	P											x	x
97	*Hyporhamphus naos* Banford & Collette, 2001	P											x	x
**XXXIII Family Belonidae**x														
98	*Strongylura hubbsi* Collette, 1974	P (V)			x	x	x	x		x	x	x		
99	*Strongylura exilis* (Girard, 1854)	P												x
100	*Tylosurus fodiator* Jordan & Gilbert, 1882	P												x
**Order Cyprinodontiformes**														
**XXXIV Family Rivulidae**														
101	*Cynodonichthys tenuis* Meek, 1904	SF				x		x		x	x	x		
**XXXV Family Profundulidae**														
102	*Profundulus punctatus* (Günther, 1866)	SF		x	x								x	
103	*Tlaloc candalarius* (Hubbs, 1924)	SF	x											
104	*Tlaloc hildebrandi* Miller, (1950)	SF					x		x					
105	*Tlaloc labialis* (Günther, 1866)	SF	x	x	x	x	x	x					x	
**XXXVI Family Anablepidae**														
106	*Anableps dowei* Gill, 1861	SF											x	x
**XXXVII Family Poeciliidae**														
107	*Belonesox belizanus* Kner, 1860	SF				x		x		x	x	x		
108	*Brachyrhaphis hartwegi* Rosen & Bailey, 1982	SF												x
109	*Carlhubbsia kidderi* (Hubbs, 1936)	SF				x					x	x		
110	*Gambusia eurystoma* Miller, 1975	SF					x							
111	*Gambusia sexradiata* Hubbs, 1936	SF		x	x	x	x	x		x	x	x		
112	*Gambusia yucatana* Regan, 1914	SF			x	x						x		
113	*Heterophallus echeagarayi* (Alvarez, 1952)	SF				x						x		
114	*Heterophallus milleri* Radda, 1987	SF					x							
115	*Phallichthys fairweatheri* Rosen & Bailey, 1959	SF								x	x	x		
116	*Poecilia kykesis* Poeser, 2002	SF										x		
117	*Poecilia mexicana* Steindachner, 1863	SF			x	x	x	x	x	x	x	x		
118	*Poecilia nelsoni* (Meek, 1904)	SF											x	x
119	*Poecilia sphenops* Valenciennes, 1836	SF	x	x	x	x		x		x	x	x	x	x
120	*Poecilia sulphuraria* (Alvarez, 1948)	SF					x							
121	*Poecilia thermalis* Steindachner, 1863	SF					x							
122	*Poeciliopsis fasciata* (Meek, 1904)	SF	x	x	x								x	x
123	*Poeciliopsis hnliickai* Meyer & Vogel, 1981	SF	x	x	x									
124	*Poeciliopsis pleurospilus* (Günther, 1868)	SF	x	x	x	x							x	x
125	*Poeciliopsis turrubarensis* (Meek, 1912)	SF											x	x
126	*Priapella intermedia* Alvarez & Carranza, 1952	SF			x									
127	*Priapella chamulae* Schartl, Meyer & Wilde, 2006	SF				x	x							
128	*Priapella compressa* Alvarez, 1948	SF						x						
129	*Priapella lacandonae* Meyer, Schories & Schartl, 2011	SF										x		
130	*Pseudoxiphophorus bimaculatus* (Heckel, 1848)	SF			x	x	x	x	x	x	x	x		
131	*Xenodexia ctenolepis* Hubbs, 1950	SF								x				
132	*Xiphophorus alvarezi* Rosen, 1960	SF							x					
133	*Xiphophorus clemenciae* Álvarez, 1959	SF			x									
134	*Xiphophorus hellerii* Heckel, 1848	SF			x	x	x	x		x	x	x		
135	*Xiphophorus maculatus* (Günther, 1866)	SF				x				x	x	x		
**Order Syngnathiformes**														
**XXXVIII Family Syngnathidae**														
136	*Hippocampus ingens* Girard, 1859	P											x	x
137	*Pseudophallus starksii* (Jordan & Culver, 1895)	P											x	x
**XXXIX Family Fistulariidae**														
138	*Fistularia commersonii* Rüppell, 1838	P											x	
**Order Synbranchiformes**														
**XL Family Synbranchidae**														
139	*Ophisternon aenigmaticum* Rosen & Greenwood, 1976	PF		x	x	x	x	x		x	x	x		
140	*Synbranchus marmoratus* Bloch, 1795	PF											x	x
**Order Perciformes**														
**XLI Family Centropomidae**														
141	*Centropomus armatus* Gill, 1863	P											x	x
142	*Centropomus medius* Günther, 1864	P											x	x
143	*Centropomus nigrescens* Günther, 1864	P											x	x
144	*Centropomus robalito* Jordan & Gilbert, 1882	P											x	x
145	*Centropomus undecimalis* (Bloch, 1792)	P				x				x	x	x		
146	*Centropomus parallelus* Poey, 1860	P								x		x		
147	*Centropomus poeyi* Chávez, 1961	P										x		
148	*Centropomus unionensis* Bocourt, 1868	P												x
149	*Centropomus viridis* Lockington, 1877	P											x	x
**XLII Family Serranidae**														
150	*Dermatolepis dermatolepis* (Boulenger, 1895)	P											x	
151	*Alphestes multiguttatus* (Günther, 1867)	P											x	x
152	*Epinephelus labriformis* (Jenyns, 1840)	P											x	
153	*Epinephelus analogus* Gill, 1863	P												x
154	*Epinephelus quinquefasciatus* (Bocourt, 1868)	P												x
155	*Mycteroperca xenarcha* Jordan, 1888	P												x
156	*Rypticus nigripinnis* Gill, 1861	P												x
**XLIII Family Centrarchidae**														
157	*Micropterus salmoides* (Lacepéde, 1802)^Ex^	Ex	x	x										
**XLIV Family Nematistiidae**														
158	*Nematistius pectoralis* Gill, 1862	P											x	x
**XLV Family Carangidae**														
159	*Carangoides otrynter* (Jordan & Gilbert, 1883)	P												x
160	*Carangoides vinctus* (Jordan & Gilbert, 1882)	P												x
161	*Caranx caballus* Günther, 1868	P												x
162	*Caranx caninus* Günther, 1867	P											x	x
163	*Caranx sexfasciatus* Quoy & Gaimard, 1825	P												x
164	*Chloroscombrus orqueta* Jordan & Gilbert, 1883	P											x	
165	*Gnathanodon speciosus* (Forsskål, 1775)	P											x	
166	*Hemicaranx leucurus* (Günther 1864)	P												x
167	*Hemicaranx zelotes* Gilbert, 1898	P											x	x
168	*Oligoplites altus* (Günther, 1868)	P											x	x
169	*Oligoplites saurus* (Bloch & Schneider, 1801)	P											x	x
170	*Selene brevoortii* (Gill, 1863)	P												x
171	*Selene oerstedii* Lütken, 1880	P											x	x
172	*Selene peruviana* (Guichenot, 1866)	P											x	x
173	*Trachinotus kennedyi* Steindachner, 1876	P											x	x
174	*Trachinotus paitensis* Cuvier, 1832	P											x	
175	*Trachinotus rhodopus* Gill, 1863	P											x	x
**XLVI Family Lutjanidae**														
176	*Hoplopagrus guentherii* Gill, 1862	P											x	x
177	*Lutjanus argentiventris* (Peters, 1869)	P											x	x
178	*Lutjanus colorado* Jordan & Gilbert, 1882	P											x	x
179	*Lutjanus guttatus* (Steindachner, 1869)	P											x	x
180	*Lutjanus novemfasciatus* Gill, 1862	P											x	x
**XLVII Family Lobotidae**														
181	*Lobotes pacificus* Gilbert, 1898	P												x
**XLVIII Family Gerreidae**														
182	*Diapterus brevirostris* (Sauvage, 1879)	P											x	x
183	*Eucinostomus currani* Zahuranec, 1980	P											x	x
184	*Eucinostomus dowii* (Gill, 1863)	P											x	x
185	*Eucinostomus gracilis* (Gill, 1862)	P											x	
186	*Eugerres axillaris* (Günther, 1864)	P											x	x
187	*Eugerres lineatus* (Humboldt, 1821)	P											x	
188	*Eugerres mexicanus* (Steindachner, 1879)	P (V)		x	x	x	x	x				x		
189	*Gerres simillimus* Regan, 1907	P											x	x
**XLIX Family Haemulidae**														
190	*Conodon serrifer* Jordan & Gilbert, 1882	P											x	
191	*Genyatremus pacifici* (Günther, 1864)	P											x	x
192	*Haemulopsis axillaris* (Steindachner, 1869)	P											x	x
193	*Haemulopsis elongatus* (Steindachner, 1879)	P											x	
194	*Haemulopsis leuciscus* (Günther, 1864)	P											x	x
195	*Haemulopsis nitidus* (Steindachner, 1869)	P											x	
196	*Orthopristis chalceus* (Günther, 1864)	P											x	
197	*Pomadasys bayanus* Jordan & Evermann, 1898	P												x
198	*Pomadasys branickii* (Steindachner, 1879)	P											x	
199	*Pomadasys macracanthus* (Günther, 1864)	P											x	x
200	*Pomadasys panamensis* (Steindachner, 1876)	P											x	
**L Family Polynemidae**														
201	*Polydactylus approximans* (Lay & Bennett, 1839)	P											x	x
202	*Polydactylus opercularis* (Gill, 1863)	P											x	x
**LI Family Sciaenidae**														
203	*Aplodinotus grunniens* Rafinesque, 1819	P (V)		x	x	x	x	x		x	x	x		
204	*Bairdiella armata* Gill, 1863	P												x
205	*Bairdiella ensifera* (Jordan & Gilbert, 1882)	P											x	x
206	*Bairdiella icistia* (Jordan & Gilbert, 1882)	P											x	
207	*Cynoscion albus* (Günther, 1864)	P											x	x
208	*Cynoscion stolzmanni* (Steindachner, 1879)	P											x	
209	*Cynoscion xanthulus* Jordan & Gilbert, 1882	P											x	
210	*Elattarchus archidium* (Jordan & Gilbert, 1882)	P											x	
211	*Isopisthus remifer* Jordan & Gilbert, 1882	P												x
212	*Larimus effulgens* Gilbert, 1898	P												x
213	*Menticirrhus elongatus* (Günther, 1864)	P												x
214	*Menticirrhus nasus* (Günther, 1868)	P											x	x
215	*Menticirrhus panamensis* (Steindachner, 1876)	P											x	
216	*Micropogonias altipinnis* (Günther, 1864)	P											x	x
217	*Micropogonias megalops* (Gilbert, 1890)	P											x	
218	*Nebris occidentalis* Vaillant, 1897	P												x
219	*Paralonchurus goodei* Gilbert, 1898	P												x
220	Stellifer cf. walkeri	P												x
**LII Family Mullidae**														
221	*Pseudupeneus grandisquamis* (Gill, 1863)	P											x	
**LIII Family Kyphosidae**														
222	*Kyphosus elegans* (Peters, 1869)	P											x	x
**LIV Family Chaetodontidae**														
223	*Chaetodon humeralis* Günther, 1860	P											x	x
**LV Family Cichlidae**														
224	*Amphilophus trimaculatus* (Günther, 1867)	SF		x	x								x	x
225	*Astatheros macracanthus* (Günther, 1864)	SF	x		x								x	x
226	*Chiapaheros grammodes* (Taylor & Miller, 1980)	SF	x	x										
227	*Cincelichthys pearsei* (Hubbs, 1936)	SF		x	x	x		x		x	x	x		
228	*Chuco intermedium* (Günther, 1862)	SF	x				x	x	x	x	x	x		
229	*Cribroheros robertsoni* (Regan, 1905)	SF				x						x		
230	*Kihnichthys ufermanni* Allgayer, 2002	SF								x	x	x		
231	*Maskaheros argenteus* (Allgayer, 1991)	SF				x				x	x	x		
232	*Maskaheros regani* (Miller, 1974)	SF		x	x									
233	*Mayaheros urophthalmus* (Günther, 1862)	SF			x	x				x	x	x		
234	*Oreochromis aureus* (Steindachner, 1864) ^Ex^	Ex								x				
235	*Oreochromis mossambicus* (Peters, 1852) ^Ex^	Ex	x		x					x				
236	*Oreochromis niloticus* (Linnaeus, 1758) ^Ex^	Ex	x	x	x	x	x	x		x	x	x	x	x
237	*Oscura heterospila* (Hubbs, 1936)	SF				x		x				x		
238	*Parachromis friedrichsthalii* (Heckel, 1840)	SF								x	x	x		
239	*Parachromis managuensis* (Günther, 1867) ^Ex^	Ex		x	x	x		x		x	x	x		x
240	*Paraneetroplus gibbiceps* (Steindachner, 1864)	SF					x	x						
241	*Petenia splendida* Günther, 1862	SF		x	x	x		x		x	x	x		
242	*Rocio ocotal* Schmitter-Soto, 2007	SF								x				
243	*Rocio octofasciata* (Regan, 1903)	SF				x		x		x	x	x		
244	*Theraps irregularis* Günther, 1862	SF							x	x	x	x		
245	*Thorichthys meeki* Brind, 1918	SF				x				x	x	x		
246	*Thorichthys pasionis* (Rivas, 1962)	SF				x					x	x		
247	*Thorichthys socolofi* (Miller & Taylor, 1984)	SF						x		x				
248	*Thorichthys helleri* (Steindachner, 1864)	SF			x	x	x	x	x	x	x	x		
249	*Trichromis salvini* (Günther, 1862)	SF			x	x		x	x	x	x	X		
250	*Tilapia zilli* (Gervais, 1848) ^Ex^	Ex	x	x	x									
251	*Rheoheros coeruleus* (Stawikowski & Werner, 1987)	SF						x						
252	*Rheoheros lentiginosus* (Steindachner, 1864)	SF					x	x	x	x	x	x		
253	*Vieja bifasciata* (Steindachner, 1864)	SF				x	x	x		x	x	x		
254	*Vieja breidohri* (Werner & Stawikowski, 1987)	SF	x											
255	*Vieja guttulata* (Günther, 1864)	SF												x
256	*Vieja hartwegi* (Taylor & Miller, 1980)	SF	x	x	x	x								
257	*Vieja melanura* (Günther, 1862)	SF		x	x	x		x		x	x	x		
258	*Wajpamheros nourissati* (Allgayer, 1989)	SF								x	x	x		
**LVI Family Pomacentridae**														
259	*Abudefduf troschelii* (Gill, 1862)	P											x	x
260	*Stegastes flavilatus* (Gill, 1862)	P											x	
**LVII Family Labridae**														
261	*Halichoeres aestuaricola* Bussing, 1972	P												x
262	*Halichoeres dispilus* (Günther, 1864)	P											x	
**LVIII Family Scaridae**														
263	*Nicholsina denticulata* (Everman & Radcliffe, 1917)	P											x	
**LIX Family Dactyloscopidae**														
264	*Dactyloscopus lunaticus* Gilbert, 1890	P											x	x
265	*Dactyloscopus amnis* Miller & Briggs, 1962	P											x	
**LX Family Eleotridae**														
266	*Dormitator latifrons* (Richardson, 1844)	P											x	x
267	*Eleotris picta* Kner, 1863	P											x	x
268	*Erotelis armiger* (Jordan & Richardson, 1895)	P											x	x
269	*Gobiomorus dormitor* Lacepéde, 1800	P				x		x		x	x	x		
270	*Gobiomorus maculatus* (Günther, 1859)	P											x	x
271	*Guavina micropus* (Ginsburg, 1953)	P												x
272	*Leptophilypnus guatemalensis* Thacker & Pezold, 2006	P (V)								x	x			
**LXI Family Gobiidae**														
273	*Aboma etheostoma* Jordan & Starks, 1895	P											x	x
274	*Awaous transandeanus* (Günther, 1861)	P (Ca)											x	
275	*Barbulifer mexicanus* Hoese & Larson, 1985	P											x	
276	*Bathygobius andrei* (Sauvage, 1880)	P											x	x
277	*Ctenogobius sagittula* (Günther, 1862)	P											x	x
278	*Evorthodus minutus* Meek & Hildebrand, 1928	P											x	x
279	*Gobioides peruanus* (Steindachner, 1880)	P												x
280	*Gobionellus liolepis* (Meek & Hildebrand, 1928)	P												x
281	*Gobionellus microdon* (Gilbert, 1892)	P											x	x
282	*Microgobius miraflorensis* Gilbert & Starks, 1904	P											x	x
283	*Parrella lucretiae* (Eigenmann & Eigenmann, 1888)	P											x	
284	*Sicydium salvini* Ogilvie-Grant, 1884	P (Ca)												x
**LXII Family Microdesmidae**														
285	*Microdesmus dorsipunctatus* Dawson, 1968	P											x	x
286	*Microdesmus suttkusi* Gilbert, 1966	P												x
**LXIII Family Ephippidae**														
287	*Chaetodipterus zonatus* (Girard, 1858)	P											x	x
288	*Parapsettus panamensis* (Steindachner, 1876)	P												x
**LXIV Family Acanthuridae**														
289	*Acanthurus xanthopterus* Valenciennes, 1835	P											x	
**LXV Family Sphyraenidae**														
290	*Sphyraena ensis*	P											x	
**LXVI Family Trichiuridae**														
291	*Trichiurus nitens* Garman, 1899	P												x
**LXVII Family Scombridae**														
292	*Scomberomorus sierra* Jordan & Starks, 1895	P											x	x
**Order Pleuronectiformes**														
**LXVIII Family Paralichthydae**														
293	*Citharichthys gilberti* Jenkins & Evermann, 1889	P											x	x
294	*Cyclopsetta panamensis* (Steindachner, 1876)	P											x	
295	*Etropus crossotus* Jordan & Gilbert, 1882	P											x	
296	*Syacium latrifons* (Jordan & Gilbert, 1882)	P											x	
297	*Syacium ovale* (Günther, 1864)	P											x	
**LXIX Family Achiridae**														
298	*Achirus mazatlanus* (Steindachner, 1869)	P											x	x
299	*Achirus scutum* (Günther, 1862)	P											x	x
300	*Achirus zebrinus* Clark, 1936	P											x	
301	*Trinectes fimbriatus* (Günther, 1862)	P												x
302	*Trinectes fonsecensis* (Günther, 1862)	P											x	x
**LXX Family Cynoglossidae**														
303	*Symphurus chabanaudi* Mahadeva & Munroe, 1990	P												x
304	*Symphurus elongatus* (Günther, 1868)	P											x	
305	*Symphurus melanurus* Clark, 1936	P											x	
**Order Tetraodontiformes**														
**LXXI Family Balistidae**														
306	*Pseudobalistes naufragium* (Jordan & Starks, 1895)	P											x	x
**LXXII Family Tetraodontidae**														
307	*Arothron meleagris* (Bloch & Schneider, 1801)	P												x
308	*Sphoeroides annulatus* (Jenyns, 1842)	P											x	x
309	*Sphoeroides rosenblatti* Bussing, 1996	P											x	x
**LXXIII Family Diodontidae**														
310	*Diodon holocanthus* Linnaeus, 1758	P												x
311	*Diodon hystrix* Linnaeus, 1758	P											x	x
**Total species by geographical units**			**23**	**31**	**45**	**55**	**36**	**46**	**11**	**63**	**54**	**72**	**174**	**153**

## Discussion

Knowledge of the species richness of continental fishes in Chiapas has increased significantly over recent years compared to previous assessments (e.g. Rodiles-Hernández et al. 2005, Velázquez-Velázquez et al. 2013). The increasing number of known species is the result of collections in new localities, improvement in sampling effort, and larger systematic and taxonomic reviews. For instance, an extensive literature search provided many reports of marine species, principally elasmobranchs, in continental waters of Chiapas by Castro-Aguirre et al. (1999). The large increment in the checklist is due to the inclusion of many elasmobranchs fishes that were included previously in the work of Castro-Aguirre et al. (1999), but that for some reason these records were ignored in more recent accounts of fishes in the continental waters of Chiapas. Castro-Aguirre et al. (1999) reported 41 species of marine fishes including an important number of sharks and sting-rays in the state continental water.

Two species previously reported were removed from the list of species in Chiapas in this study: the American eel (*Anguilla rostrata*) and the Mexican tetra (*Astyanax mexicanus*). The American eel was mentioned in the pioneering work of Velasco-Colín (1976), and since then listed in subsequent publications (Lozano-Vilano and Contreras-Balderas 1987, Rodiles-Hernández 2005, Rodiles-Hernández et al. 2005, Espinosa-Pérez et al. 2011, Velázquez-Velázquez et al. 2013). However, these works do not offer precise geographical locations for these species and there are no vouchered specimens from Chiapas in national or international collections. Records of the Mexican tetra in Chiapas probably contain misidentifications as mentioned by Lozano-Vilano and Contreras-Balderas (1987) and Ornelas-García et al. (2008), thus supporting the absence of this species in Southern Mexico. We have included Important and recent taxonomic changes made in the family Cichlidae by McMahan et al. (2015) and Říčan et al. (2016), the family Poeciliidae by Palacios et al. (2016) and the family Profundulidae by Morcillo et al. (2016).

More than 1000 species of fishes have been reported in the continental waters of Mexico, including freshwater and estuarine fishes (Espinosa-Pérez 2014). The continental fish fauna of the state of Chiapas represents approximately 29% of the continental fish fauna of the entire country of Mexico. This highlights the great diversity of fishes inhabiting continental environments of Chiapas as a result of the region’s hydrological wealth. Our results are comparable with those from other southern Mexican states such as Quintana Roo (Schmitter-Soto 1998), Oaxaca (Martínez-Ramírez et al. 2004) and Tabasco (Espinosa-Pérez and Daza-Zepeda 2005).

The native obligate freshwater (primary and secondary) species of Chiapas accounted for only 26% (81) of the state’s total species richness. The communities are dominated by peripheral species, many of them permanent (vicarious) residents of the Grijalva-Usumacinta basin (e.g. *Aplodinotus grunniens*, *Eugerres mexicanus*, *Hyporhamphus mexicanus*, *Strongylura hubbsi*), but the majority are distributed in brackish environments of the Costa-Itsmo and Costa-Soconusco sub-basins. Some of these communities also permeate nearby rivers. In terms of slopes, the Pacific slope houses 68% of the state fish fauna while the Gulf slope houses 33%, and in terms of regional diversity the Usumacinta region is considered one of the most diverse areas of endemism for freshwater fishes in Central America; however, from a biogeographical perspective the entire Central American region has a depauperate freshwater fish fauna compared with the vast diversity of ostariophysan fishes found in North and South America (Miller 1966, Myers 1966, Bussing 1985, Chakrabarty and Albert 2011, Matamoros et al. 2015). This could explain the presence of a great number of peripheral species recorded in the continental environments of Chiapas. This pattern is comparable with other countries of Central America such as Guatemala (Kihn-Pineda et al., 2006), Honduras (Matamoros et al. 2009) and El Salvador (McMahan et al. 2013).

Mexican law protects thirteen freshwater species; however, *Rhamdia guatemalensis* is quite abundant in Chiapas and possesses a wide distribution through other geographic areas of Mexico and Central America (Miller et al. 2005, Hernández et al. 2015). Its inclusion should be reconsidered in the NOM-059-SEMARNAT-2010. Conversely, we suggest that Mexican laws should consider including *Lacantunia enigmatica*, *Rhamdia laluchensis* and *Vieja breidohri* as protected species on the grounds of their restricted distribution.

Since the pioneering work of Lozano-Vilano and Contreras-Balderas (1987), this is the first time the state of Chiapas has been regionalized in a more detailed scale than the three great basins (Grijalva, Usumacinta and Costa). Lozano-Vilano and Contreras-Balderas (1987) proposed seven physiographic regions; however, their proposal was based on physiographic characteristics of landscape relief rather than hydrology. In this study we present a zonation based on the level of hydrological regions (sub-basins), which provides a more robust delineation of the geographical areas for fish species and facilitates a closer examination of the distribution of endemic species. This approach demonstrates that gaps in knowledge of the distribution of species is still quite large and indicates that some portions of the territory remain moderately sampled or unexplored. For instance, the Usumacinta-Jataté sub-basin, with only 11 species recorded, remains largely unexplored. The detailed regionalization of Chiapas highlights the necessity of increasing sampling efforts in certain zones.

Although hydrological regions Grijalva, Usumacinta and Costa of Chiapas have been used in previous studies to discover endemism in the state (Rodiles-Hernández 2005, Rodiles-Hernández et al. 2005, Velázquez-Velázquez et al. 2013), the zonation of our study allows identification of smaller geographic units, permitting us to be more specific in studies of endemism. Thus, the distribution of endemic species in Chiapas includes: *Lacantunia enigmatica* in Usumacinta-Lacantún, *Rocio ocotal* in Usumacinta-Lacantún, *Thorichthys socolofi* in Grijalva-Tulijá and Usumacinta-Lacantún, *Tlaloc hildebrandi* in Grijalva-Teapa and Usumacinta-Jataté, and *Poecilia thermalis* in Grijalva-Teapa. Of the 12 units, Usumacinta-Lacantún stands out as it houses three endemic species: *Lacantunia enigamatica*, *Rocio ocotal*, and *Thorichthys socolofi*.

Forty years of scientific research on the continental fish fauna of Chiapas has gone a long way since the work of Velasco-Colín (1976). However, this does not seem nearly enough time to completely finish to record the real extend of the state species richness with its distribution. In this work we present distributional data at 12 geographic units. However, although this is the finest distributional scale for the state, a major goal should be to complete distributional data for the 92 existing sub-drainages in the state. Many of these water bodies have never been sampled either for lack of financial resources or because they are located in remote areas of the state.
